# Supplementary material for: First Detection of Meloidogyne luci (Nematoda: Meloidogynidae) Parasitizing Potato in the Azores, Portugal
Source: Plants (Basel). 2021 Jan 6;10(1):99. doi: 10.3390/plants10010099 (PMC7825027; doi:10.3390/plants10010099)
Supplement: Supplementary file 1 [file plants-10-00099-s001.pdf]

Supplementary material

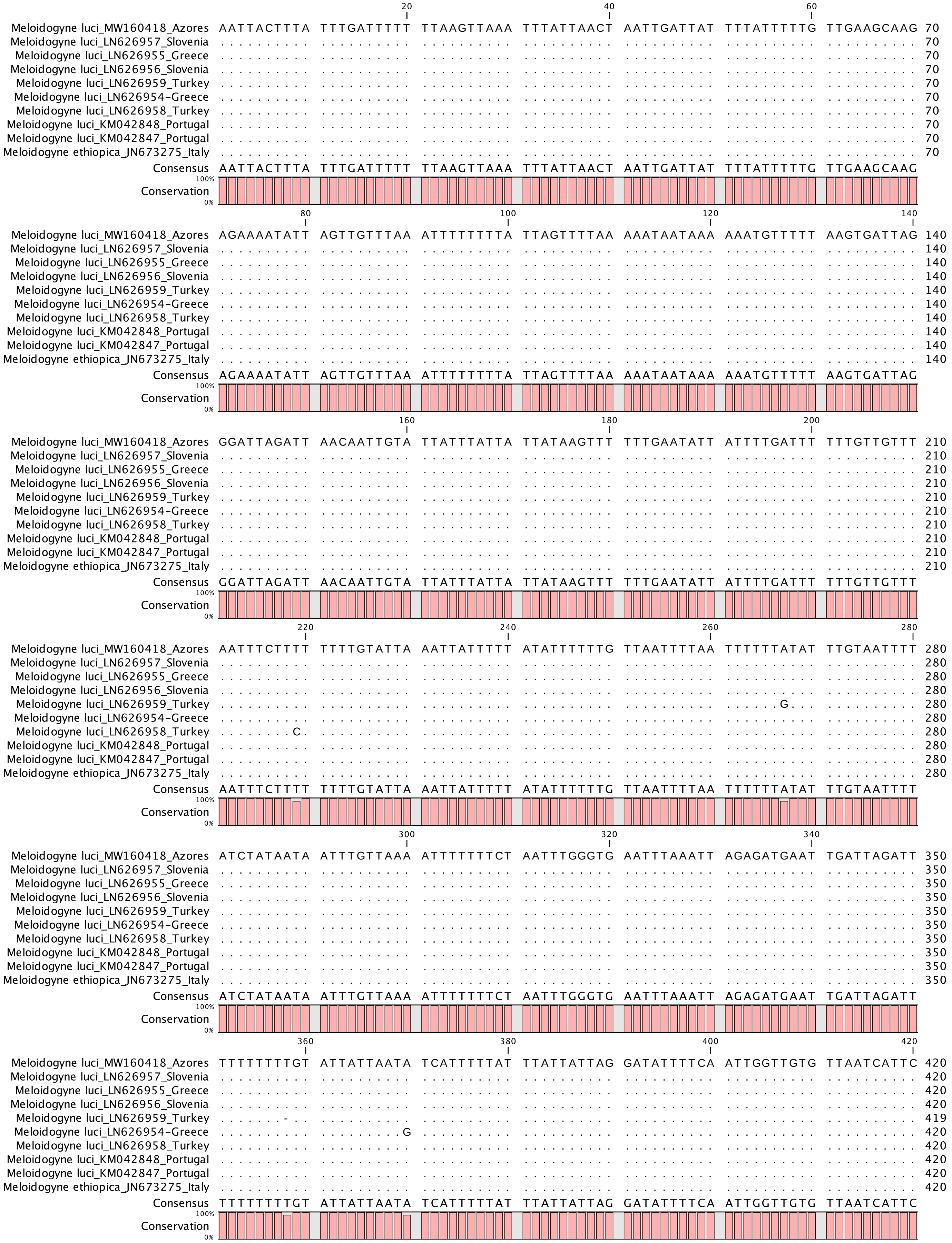

```
                                                             440                 460                 480
Meloidogyne luci_MW160418_Azores       TTTATTAGAT CGGGGTTTAA TAATGGGTTC TTTATTGGGT TAATTATTAA AATATTATTT TGGTTTTTTA 490
Meloidogyne luci_LN626957_Slovenia     .......... .......... .......... .......T.. .......... .......... .......... 490
Meloidogyne luci_LN626955_Greece       .......... .......... .......... .......T.. .......... .......... .......... 490
Meloidogyne luci_LN626956_Slovenia     .......... .......... .......... .......T.. .......... .......... .......... 490
Meloidogyne luci_LN626959_Turkey       .......... .......... .......... .......T.. .......... .......... .......... 489
Meloidogyne luci_LN626954-Greece       .......... .......... .......... .......T.. .......... .......... .......... 490
Meloidogyne luci_LN626958_Turkey       .......... .......... .......... .......T.. .......... .......... .......... 490
Meloidogyne luci_KM042848_Portugal     .......... .......... .......... .......T.. .......... .......... .......... 490
Meloidogyne luci_KM042847_Portugal     .......... .......... .......... .......T.. .......... .......... .......... 490
Meloidogyne ethiopica_JN673275_Italy   .......... .......... .......... .......T.. .......... .......... .......... 490
Consensus                              TTTATTAGAT CGGGGTTTAA TAATGGGTTC TTTATTGTGT TAATTATTAA AATATTATTT TGGTTTTTTA
Conservation (100% / 0%)

                                                   500                 520                 540                 560
Meloidogyne luci_MW160418_Azores       GTGAAATTTT TTATTTAATT TATTTTTATA CGAAAAATTA TTAAGGAGAA AATTTTGATT TTTTGTTATT 560
Meloidogyne luci_LN626957_Slovenia     .......... .......... .......... .......... .......... G......... .......... 560
Meloidogyne luci_LN626955_Greece       .......... .......... .......... .......... .......... G......... .......... 560
Meloidogyne luci_LN626956_Slovenia     .......... .......... .......... .......... .......... G......... .......... 560
Meloidogyne luci_LN626959_Turkey       .......... .......... .......... .......... .......... G......... .......... 559
Meloidogyne luci_LN626954-Greece       .......... .......... .......... .......... .......... G......... .......... 560
Meloidogyne luci_LN626958_Turkey       .......... .......... .......... .......... .......... G......... .......... 560
Meloidogyne luci_KM042848_Portugal     .......... .......... .......... .......... .......... G......... .......... 560
Meloidogyne luci_KM042847_Portugal     .......... .......... .......... .......... .......... G......... .......... 560
Meloidogyne ethiopica_JN673275_Italy   .......... .......... .......... .......... .......... G......... .......... 560
Consensus                              GTGAAATTTT TTATTTAATT TATTTTTATA CGAAAAATTA TTAAGGAGAA GATTTTGATT TTTTGTTATT
Conservation (100% / 0%)

                                                             580                 600                 620
Meloidogyne luci_MW160418_Azores       TAAAGTTTAA GTTCTTTATA GAACAAAATT TTTTATCTTT TTAGTATTCT ATTGAAATAG AAGAATTATA 630
Meloidogyne luci_LN626957_Slovenia     ..G....... .......... .......... .......... .......... .......... .......... 630
Meloidogyne luci_LN626955_Greece       ..G....... .......... .......... .......... .......... .......... .......... 630
Meloidogyne luci_LN626956_Slovenia     ..G....... .......... .......... .......... .......... .......... .......... 630
Meloidogyne luci_LN626959_Turkey       ..G....... .......... .......... .......... .......... .......... .......... 629
Meloidogyne luci_LN626954-Greece       ..G....... .......... .......... .......... .......... .......... .......... 630
Meloidogyne luci_LN626958_Turkey       ..G....... .......... .......... .......... .......... .......... T......... 630
Meloidogyne luci_KM042848_Portugal     ..G....... .......... .......... .......... .......... .......... .......... 630
Meloidogyne luci_KM042847_Portugal     ..G....... .......... .......... .......... .......... .......... .......... 630
Meloidogyne ethiopica_JN673275_Italy   ..G....... .......... .......... .......... .......... .......... .......... 630
Consensus                              TAGAGTTTAA GTTCTTTATA GAACAAAATT TTTTATCTTT TTAGTATTCT ATTGAAATAG AAGAATTATA
Conservation (100% / 0%)

                                                   640                 660                 680                 700
Meloidogyne luci_MW160418_Azores       AATTAGTTAA TTCTAATTTT ATTTATTTTA TATGGATTAT ATAAAATTTT AAATTTTTTG ATTTTAGTTT 700
Meloidogyne luci_LN626957_Slovenia     .......... .......... .......... .......... .......... .......... .......... 700
Meloidogyne luci_LN626955_Greece       .......... .......... .......... .......... .......... .......... .......... 700
Meloidogyne luci_LN626956_Slovenia     .......... .......... .......... .......... .......... .......... .......... 700
Meloidogyne luci_LN626959_Turkey       .......... .......... .......... .......... .......... .......... .......... 699
Meloidogyne luci_LN626954-Greece       .......... .......... .......... .......... .......... .......... .......... 700
Meloidogyne luci_LN626958_Turkey       .......... .......... .......... .......... .......... .......... .......... 700
Meloidogyne luci_KM042848_Portugal     .......... .......... .......... .......... .......... .......... .......... 700
Meloidogyne luci_KM042847_Portugal     .......... .......... .......... .......... .......... .......... .......... 700
Meloidogyne ethiopica_JN673275_Italy   .......... .......... .......... .......... .......... .......... .......... 700
Consensus                              AATTAGTTAA TTCTAATTTT ATTTATTTTA TATGGATTAT ATAAAATTTT AAATTTTTTG ATTTTAGTTT
Conservation (100% / 0%)

                                                             720                 740                 760
Meloidogyne luci_MW160418_Azores       ATTTACGTAT TTAGTTGATT TTTTTAATAG ATTTAGTTCA TCTGTTGATG TAACACAGAG TGGCTCTTTA 770
Meloidogyne luci_LN626957_Slovenia     .......... .......... .......... .......... .......... .......... .......... 770
Meloidogyne luci_LN626955_Greece       .......... .......... .......... .......... .......... .......... .......... 770
Meloidogyne luci_LN626956_Slovenia     .......... .......... .......... .......... .......... .......... .......... 770
Meloidogyne luci_LN626959_Turkey       .......... .......... .......... .......... .......... .......... .......... 769
Meloidogyne luci_LN626954-Greece       .......... .......... .......... .......... .......... .......... .......... 770
Meloidogyne luci_LN626958_Turkey       .......... .......... .......... .......... .......... .......... .......... 770
Meloidogyne luci_KM042848_Portugal     .......... .......... .......... .......... .......... .......... .......... 770
Meloidogyne luci_KM042847_Portugal     .......... .......... .......... .......... .......... .......... .......... 770
Meloidogyne ethiopica_JN673275_Italy   .......... .......... .......... .......... .......... .......... .......... 770
Consensus                              ATTTACGTAT TTAGTTGATT TTTTTAATAG ATTTAGTTCA TCTGTTGATG TAACACAGAG TGGCTCTTTA
Conservation (100% / 0%)

                                                   780                 800                 820                 840
Meloidogyne luci_MW160418_Azores       GTTAATTTAC CTGAAGTTAA AAATGGTCAA TTATATGATA TAACTTTTGG TTATCATCAA GAATATTTCA 840
Meloidogyne luci_LN626957_Slovenia     .......... .......... .......... .......... .......... .......... .......... 840
Meloidogyne luci_LN626955_Greece       .......... .......... .......... .......... .......... .......... .......... 840
Meloidogyne luci_LN626956_Slovenia     .......... .......... .......... .......... .......... .......... .........G 840
Meloidogyne luci_LN626959_Turkey       .......... .......... .......... .......... .......... .......... .......... 839
Meloidogyne luci_LN626954-Greece       .......... .......... .......... .......... .......... .......... .......... 840
Meloidogyne luci_LN626958_Turkey       .......... .......... .......... .......... .......... .......... .......... 840
Meloidogyne luci_KM042848_Portugal     .......... .......... .......... .......... .......... .......... .......... 840
Meloidogyne luci_KM042847_Portugal     .......... .......... .......... .......... .......... .......... .......... 840
Meloidogyne ethiopica_JN673275_Italy   .......... .......... .......... .......... .......... .......... .......... 840
Consensus                              GTTAATTTAC CTGAAGTTAA AAATGGTCAA TTATATGATA TAACTTTTGG TTATCATCAA GAATATTTCA
Conservation (100% / 0%)
```

```
                                                     860                 880                 900
                                                       |                   |                   |
Meloidogyne luci_MW160418_Azores      AGGAAATTAT GGTGCTCATC CTGATAAAGA TCGTAATTTT TATAGTTGTG ACATTGTTAT ATGTCAATCA  910
Meloidogyne luci_LN626957_Slovenia    .......... .......... .......... .......... .......... .T........ ..........  910
Meloidogyne luci_LN626955_Greece      .......... .......... .......... .......... .......... .T........ ..........  910
Meloidogyne luci_LN626956_Slovenia    .......... .......... .......... .......... .......... .T........ ..........  910
Meloidogyne luci_LN626959_Turkey      .......... .......... .......... .......... .......... .T........ ..........  909
Meloidogyne luci_LN626954-Greece      .......... .......... .......... .......... .......... .T........ ..........  910
Meloidogyne luci_LN626958_Turkey      .......... .......... .......... .......... .......... .T........ ..........  910
Meloidogyne luci_KM042848_Portugal    .......... .......... .......... .......... .......... .T........ ..........  910
Meloidogyne luci_KM042847_Portugal    .......... .......... .......... .......... .......... .T........ ..........  910
Meloidogyne ethiopica_JN673275_Italy  .......... .......... .......... .......... .......... .T........ ..........  910
                           Consensus  AGGAAATTAT GGTGCTCATC CTGATAAAGA TCGTAATTTT TATAGTTGTG ATATTGTTAT ATGTCAATCA
                                100%
                        Conservation
                                  0%

                                           920                 940                 960                 980
                                             |                   |                   |                   |
Meloidogyne luci_MW160418_Azores      CAAGTTT-AG AAGAATATCA TAATGAATTA AATTAATCTG TTAGTGAAAA AATAGTTATT TTTATTAATA  979
Meloidogyne luci_LN626957_Slovenia    .......T.. .......... .......... .......... .......... .......... ..........  980
Meloidogyne luci_LN626955_Greece      .......T.. .......... .......... .......... .......... .......... ..........  980
Meloidogyne luci_LN626956_Slovenia    .......T.. .......... .......... .......... .......... .......... ..........  980
Meloidogyne luci_LN626959_Turkey      .......T.. .......... .......... .......... .......... .......... ..........  979
Meloidogyne luci_LN626954-Greece      .......T.. .......... .......... .......... .......... .......... ..........  980
Meloidogyne luci_LN626958_Turkey      .......T.. .......... .......... .......... .......... .......... ..........  980
Meloidogyne luci_KM042848_Portugal    .......T.. .......... .......... .......... .......... .......... ..........  980
Meloidogyne luci_KM042847_Portugal    .......T.. .......... .......... .......... .......... .......... ..........  980
Meloidogyne ethiopica_JN673275_Italy  .......T.. .......... .......... .......... .......... .......... ..........  980
                           Consensus  CAAGTTTTAG AAGAATATCA TAATGAATTA AATTAATCTG TTAGTGAAAA AATAGTTATT TTTATTAATA
                                100%
                        Conservation
                                  0%

                                                   1,000               1,020               1,040
                                                       |                   |                   |
Meloidogyne luci_MW160418_Azores      GAAATATAAG GAGATTTAAT TTTTAAGTTA AATCCAATTC GTAATGTTTG GAATTTACCA AGGTAAAATT  1049
Meloidogyne luci_LN626957_Slovenia    .......... .......... .......... ....T..... .......... .......... .....G....  1050
Meloidogyne luci_LN626955_Greece      .......... .......... .......... ....T..... .......... .......... .....G....  1050
Meloidogyne luci_LN626956_Slovenia    .......... .......... .......... ....T..... .......... .......... .....G....  1050
Meloidogyne luci_LN626959_Turkey      .......... .......... .......... ....T..... .......... .......... .....G....  1049
Meloidogyne luci_LN626954-Greece      .......... .......... .......... ....T..... .......... .......... .....G....  1050
Meloidogyne luci_LN626958_Turkey      .......... .......... .......... ....T..... .......... .......... .....G....  1050
Meloidogyne luci_KM042848_Portugal    .......... .......... .......... ....T..... .......... .......... .....G....  1050
Meloidogyne luci_KM042847_Portugal    .......... .......... .......... ....T..... .......... .......... .....G....  1050
Meloidogyne ethiopica_JN673275_Italy  .......... .......... .......... ....T..... .......... .......... .....G....  1050
                           Consensus  GAAATATAAG GAGATTTAAT TTTTAAGTTA AATCTAATTC GTAATGTTTG GAATTTACCA AGGTAGAATT
                                100%
                        Conservation
                                  0%

                                         1,060               1,080               1,100               1,120
                                             |                   |                   |                   |
Meloidogyne luci_MW160418_Azores      ACACGTTAAA TTTAGAAGAA TTGTTGAAAA GAATGAATTT TTAATGGAAA CAGTAAGGAT ATTTTTTATA  1119
Meloidogyne luci_LN626957_Slovenia    .......... .......... .......... .........C .......... .......... ..........  1120
Meloidogyne luci_LN626955_Greece      .......... .......... .......... .........C .......... .......... ..........  1120
Meloidogyne luci_LN626956_Slovenia    .......... .......... .......... .........C .......... .......... ..........  1120
Meloidogyne luci_LN626959_Turkey      .......... .......... .......... .........C .......... .......... ..........  1119
Meloidogyne luci_LN626954-Greece      .......... .......... .......... .........C .......... .......... ..........  1120
Meloidogyne luci_LN626958_Turkey      .......... .......... .......... .........C .......... .......... ..........  1120
Meloidogyne luci_KM042848_Portugal    .......... .......... .......... .........C .......... .......... ..........  1120
Meloidogyne luci_KM042847_Portugal    .......... .......... .......... .G.......C .......... .......... ..........  1120
Meloidogyne ethiopica_JN673275_Italy  .......... .......... .......... .........C .......... .......... ..........  1120
                           Consensus  ACACGTTAAA TTTAGAAGAA TTGTTGAAAA GAATGAATTC TTAATGGAAA CAGTAAGGAT ATTTTTTATA
                                100%
                        Conservation
                                  0%

                                                   1,140               1,160               1,180
                                                       |                   |                   |
Meloidogyne luci_MW160418_Azores      TAATTATTTT TAATAATATT AAAAATAAAG CTATTAATTT CAATTGAATT TTTTATTGTG ATTAAAAAAG  1189
Meloidogyne luci_LN626957_Slovenia    .......... .......... .......... .......... .......... .......... ..........  1190
Meloidogyne luci_LN626955_Greece      .......... .......... .......... .......... .......... .......... ..........  1190
Meloidogyne luci_LN626956_Slovenia    .......... .......... .......... .......... .......... .......... ..........  1190
Meloidogyne luci_LN626959_Turkey      .......... .....T.... .......... .......... .......... .......... ..........  1189
Meloidogyne luci_LN626954-Greece      .......... .......... .......... .......... .......... .......... ..........  1190
Meloidogyne luci_LN626958_Turkey      .......... .....T.... .......... .......... .......... .......... ..........  1190
Meloidogyne luci_KM042848_Portugal    .......... .......... .......... .......... .......... .......... ..........  1190
Meloidogyne luci_KM042847_Portugal    .......... .......... .......... .......... .......... .......... ..........  1190
Meloidogyne ethiopica_JN673275_Italy  .......... .......... .......... .......... .......... .......... ..........  1190
                           Consensus  TAATTATTTT TAATAATATT AAAAATAAAG CTATTAATTT CAATTGAATT TTTTATTGTG ATTAAAAAAG
                                100%
                        Conservation
                                  0%

                                         1,200               1,220               1,240               1,260
                                             |                   |                   |                   |
Meloidogyne luci_MW160418_Azores      TTTTTGGCTA AATTATTTTT TTAGATTATT TTTTATTGTT GAAAAAATTA AAAACAAATT GTTTTTTACA  1259
Meloidogyne luci_LN626957_Slovenia    .......... .......... .......... .......... .......... .......... ..........  1260
Meloidogyne luci_LN626955_Greece      .......... .......... .......... .......... .......... .......... ..........  1260
Meloidogyne luci_LN626956_Slovenia    .......... .......... .......... .......... .......... .......... ..........  1260
Meloidogyne luci_LN626959_Turkey      .......... .......... .......... .......... .......... .......... ..........  1259
Meloidogyne luci_LN626954-Greece      .......... .......... .......... .......... .......... .......... ..........  1260
Meloidogyne luci_LN626958_Turkey      .......... .......... .......... .......... .......... .......... ..........  1260
Meloidogyne luci_KM042848_Portugal    .......... .......... .......... .......... .......... .......... ......-...  1259
Meloidogyne luci_KM042847_Portugal    .......... .......... .......... .......... .......... .......... ..........  1260
Meloidogyne ethiopica_JN673275_Italy  .......... .......... .......... .......... .......... .......... ..........  1260
                           Consensus  TTTTTGGCTA AATTATTTTT TTAGATTATT TTTTATTGTT GAAAAAATTA AAAACAAATT GTTTTTTACA
                                100%
                        Conservation
                                  0%
```

```
                                          1,280                1,300                1,320
                                            |                    |                    |
Meloidogyne luci_MW160418_Azores    ATAATTAAAA TTTATAATAT TTCAATTTTT AATTTTTAGT TTTAAATAAA AATTACAAAT ATGAAAAATA 1329
Meloidogyne luci_LN626957_Slovenia  .......... .......... .......... .......... .......... .......... .......... 1330
Meloidogyne luci_LN626955_Greece    .......... .......... .......... .......... .......... .......... .......... 1330
Meloidogyne luci_LN626956_Slovenia  .......... .......... .......... .......... .......... .......... .......... 1330
Meloidogyne luci_LN626959_Turkey    .......... .......... .......... .......... .......... .......... .......... 1329
Meloidogyne luci_LN626954-Greece    .......... .......... .......... .......... .......... .......... .......... 1330
Meloidogyne luci_LN626958_Turkey    .......... .......... .......... .......... .......... .......... .......... 1330
Meloidogyne luci_KM042848_Portugal  .......... .......... .......... .......... .......... .......... .......... 1329
Meloidogyne luci_KM042847_Portugal  .......... .......... .......... .......... .......... .......... .......... 1330
Meloidogyne ethiopica_JN673275_Italy .......... .......... .......... .......... .......... .......... .......... 1330
Consensus                           ATAATTAAAA TTTATAATAT TTCAATTTTT AATTTTTAGT TTTAAATAAA AATTACAAAT ATGAAAAATA
Conservation (100% / 0%)

                          1,340                1,360                1,380                1,400
                            |                    |                    |                    |
Meloidogyne luci_MW160418_Azores    AAAAAATTTT GTTATAAATT AAATTAATTT ATTTATATTA AAAATATTTT TTATAATTTT TATTTTTTTT 1399
Meloidogyne luci_LN626957_Slovenia  .......... .......... .......... .......... .......... .......... .......... 1400
Meloidogyne luci_LN626955_Greece    .......... .......... .......... .......... .......... .......... .......... 1400
Meloidogyne luci_LN626956_Slovenia  .......... .......... .......... .......... .......... .......... .......... 1400
Meloidogyne luci_LN626959_Turkey    .......... .......... .......... .......... .......... .......... .......... 1399
Meloidogyne luci_LN626954-Greece    .......... .......... .......... .......... .......... .......... .......... 1400
Meloidogyne luci_LN626958_Turkey    .......... .......... .......... .......... .......... .......... .......... 1400
Meloidogyne luci_KM042848_Portugal  .......... .......... .......... .......... .......... .......... .......... 1399
Meloidogyne luci_KM042847_Portugal  .......... .......... .......... .......... .......... .......... .......... 1400
Meloidogyne ethiopica_JN673275_Italy .......... .......... .......... .......... .......... .......... .......... 1400
Consensus                           AAAAAATTTT GTTATAAATT AAATTAATTT ATTTATATTA AAAATATTTT TTATAATTTT TATTTTTTTT
Conservation (100% / 0%)

                                          1,420                1,440                1,460
                                            |                    |                    |
Meloidogyne luci_MW160418_Azores    ATTTTTTTAA AAAAAAAATA ATTTAATTAT TATTATTTAT TTAAATTTAA TAATTAAATA TAAAATTTTT 1469
Meloidogyne luci_LN626957_Slovenia  .......... .......... .......... A......... .......... .......... .......... 1470
Meloidogyne luci_LN626955_Greece    .......... .......... .......... A......... .......... .......... .......... 1470
Meloidogyne luci_LN626956_Slovenia  .......... .......... .......... A......... .......... .......... .......... 1470
Meloidogyne luci_LN626959_Turkey    .......... .......... .......... A......... .......... .......... .......... 1469
Meloidogyne luci_LN626954-Greece    .......... .......... .......... AG........ .......... .......... .......... 1470
Meloidogyne luci_LN626958_Turkey    .......... .......... .......... A......... .......... .......... .......... 1470
Meloidogyne luci_KM042848_Portugal  .......... .......... .......... A......... .......... .......... .......... 1469
Meloidogyne luci_KM042847_Portugal  .......... .......... .......... A......... .......... .......... .......... 1470
Meloidogyne ethiopica_JN673275_Italy .......... .......... .......... A......... .......... .......... .......... 1470
Consensus                           ATTTTTTTAA AAAAAAAATA ATTTAATTAT AATTATTTAT TTAAATTTAA TAATTAAATA TAAAATTTTT
Conservation (100% / 0%)

                          1,480                1,500                1,520                1,540
                            |                    |                    |                    |
Meloidogyne luci_MW160418_Azores    TATTAAATAA ATTTAATAAT AAATGTTTTT TAAATTCTTT GAGGTTTTGA TTTTTGATTT TT-GTTTCTG 1538
Meloidogyne luci_LN626957_Slovenia  .......... ........G. .......... .......... .......... .......... ..T....... 1540
Meloidogyne luci_LN626955_Greece    .......... ........G. .......... .......... .......... .......... ..T....... 1540
Meloidogyne luci_LN626956_Slovenia  .......... ........G. .......... .......... .......... .......... ..T....... 1540
Meloidogyne luci_LN626959_Turkey    .......... ........G. .......... .......... .......... .......... ..T....... 1539
Meloidogyne luci_LN626954-Greece    .......... ........G. .......... .......... .......... .......... ..T....... 1540
Meloidogyne luci_LN626958_Turkey    .......... ........G. .......... .......... .......... .......... ..T....... 1540
Meloidogyne luci_KM042848_Portugal  .......... ........G. .......... .......... .......... .......... ..T....... 1539
Meloidogyne luci_KM042847_Portugal  .......... ........G. .......... .......... .......... .......... ..T....... 1540
Meloidogyne ethiopica_JN673275_Italy .......... ........G. .......... .......... .......... .......... ..T....... 1540
Consensus                           TATTAAATAA ATTTAATAGT AAATGTTTTT TAAATTCTTT GAGGTTTTGA TTTTTGATTT TTTGTTTCTG
Conservation (100% / 0%)

Meloidogyne luci_MW160418_Azores    CTCATT--TA AAAA 1550
Meloidogyne luci_LN626957_Slovenia  ......GT.. ..G. 1554
Meloidogyne luci_LN626955_Greece    ......GT.. ..G. 1554
Meloidogyne luci_LN626956_Slovenia  ......GT.. ..G. 1554
Meloidogyne luci_LN626959_Turkey    ......GT.. ..G. 1553
Meloidogyne luci_LN626954-Greece    ......GT.. ..G. 1554
Meloidogyne luci_LN626958_Turkey    ......GT.. ..G. 1554
Meloidogyne luci_KM042848_Portugal  ......GT.. ..G. 1553
Meloidogyne luci_KM042847_Portugal  ......GT.. ..G. 1554
Meloidogyne ethiopica_JN673275_Italy ......GT.. ..G. 1554
Consensus                           CTCATTGTTA AAGA
Conservation (100% / 0%)
```

Figure S1: Alignment of M. luci isolate from Azores Island and available sequences on GenBank.
